# Comparative effectiveness of sotrovimab and molnupiravir for preventing severe COVID-19 outcomes in patients on kidney replacement therapy: observational study using the OpenSAFELY-UKRR and SRR databases

**DOI:** 10.1093/ckj/sfad184

**Published:** 2023-08-29

**Authors:** Bang Zheng, Jacqueline Campbell, Edward J Carr, John Tazare, Linda Nab, Viyaasan Mahalingasivam, Amir Mehrkar, Shalini Santhakumaran, Retha Steenkamp, Fiona Loud, Susan Lyon, Miranda Scanlon, William J Hulme, Amelia C A Green, Helen J Curtis, Louis Fisher, Edward Parker, Ben Goldacre, Ian Douglas, Stephen Evans, Brian MacKenna, Samira Bell, Laurie A Tomlinson, Dorothea Nitsch, OpenSAFELY Collaborative and LH&W NCS (or CONVALESCENCE) Collaborative

**Affiliations:** London School of Hygiene and Tropical Medicine, Keppel Street, London, UK; Scottish Renal Registry, Scottish Health Audits, Public Health Scotland, Glasgow, UK; Francis Crick Institute, London, UK; London School of Hygiene and Tropical Medicine, Keppel Street, London, UK; Bennett Institute for Applied Data Science, Nuffield Department of Primary Care Health Sciences, University of Oxford, Oxford, UK; London School of Hygiene and Tropical Medicine, Keppel Street, London, UK; Bennett Institute for Applied Data Science, Nuffield Department of Primary Care Health Sciences, University of Oxford, Oxford, UK; UK Renal Registry, Bristol, UK; UK Renal Registry, Bristol, UK; Kidney Care UK, Alton, UK; Patient Council, UK Kidney Association, Bristol, UK; Kidney Research UK, Peterborough, UK; Bennett Institute for Applied Data Science, Nuffield Department of Primary Care Health Sciences, University of Oxford, Oxford, UK; Bennett Institute for Applied Data Science, Nuffield Department of Primary Care Health Sciences, University of Oxford, Oxford, UK; Bennett Institute for Applied Data Science, Nuffield Department of Primary Care Health Sciences, University of Oxford, Oxford, UK; Bennett Institute for Applied Data Science, Nuffield Department of Primary Care Health Sciences, University of Oxford, Oxford, UK; London School of Hygiene and Tropical Medicine, Keppel Street, London, UK; Bennett Institute for Applied Data Science, Nuffield Department of Primary Care Health Sciences, University of Oxford, Oxford, UK; London School of Hygiene and Tropical Medicine, Keppel Street, London, UK; London School of Hygiene and Tropical Medicine, Keppel Street, London, UK; Bennett Institute for Applied Data Science, Nuffield Department of Primary Care Health Sciences, University of Oxford, Oxford, UK; Scottish Renal Registry, Scottish Health Audits, Public Health Scotland, Glasgow, UK; Division of Population Health and Genomics, School of Medicine, University of Dundee, Dundee, UK; London School of Hygiene and Tropical Medicine, Keppel Street, London, UK; London School of Hygiene and Tropical Medicine, Keppel Street, London, UK; UK Renal Registry, Bristol, UK

**Keywords:** cohort studies, comparative effectiveness research, COVID-19, renal replacement therapy

## Abstract

**Background:**

Due to limited inclusion of patients on kidney replacement therapy (KRT) in clinical trials, the effectiveness of coronavirus disease 2019 (COVID-19) therapies in this population remains unclear. We sought to address this by comparing the effectiveness of sotrovimab against molnupiravir, two commonly used treatments for non-hospitalised KRT patients with COVID-19 in the UK.

**Methods:**

With the approval of National Health Service England, we used routine clinical data from 24 million patients in England within the OpenSAFELY-TPP platform linked to the UK Renal Registry (UKRR) to identify patients on KRT. A Cox proportional hazards model was used to estimate hazard ratios (HRs) of sotrovimab versus molnupiravir with regards to COVID-19-related hospitalisations or deaths in the subsequent 28 days. We also conducted a complementary analysis using data from the Scottish Renal Registry (SRR).

**Results:**

Among the 2367 kidney patients treated with sotrovimab (*n* = 1852) or molnupiravir (*n* = 515) between 16 December 2021 and 1 August 2022 in England, 38 cases (1.6%) of COVID-19-related hospitalisations/deaths were observed. Sotrovimab was associated with substantially lower outcome risk than molnupiravir {adjusted HR 0.35 [95% confidence interval (CI) 0.17–0.71]; *P* = .004}, with results remaining robust in multiple sensitivity analyses. In the SRR cohort, sotrovimab showed a trend toward lower outcome risk than molnupiravir [HR 0.39 (95% CI 0.13–1.21); *P* = .106]. In both datasets, sotrovimab had no evidence of an association with other hospitalisation/death compared with molnupiravir (HRs ranged from 0.73 to 1.29; *P* > .05).

**Conclusions:**

In routine care of non-hospitalised patients with COVID-19 on KRT, sotrovimab was associated with a lower risk of severe COVID-19 outcomes compared with molnupiravir during Omicron waves.

## INTRODUCTION

People receiving kidney replacement therapy (KRT) remain vulnerable to severe outcomes from COVID-19 [1]. This is multifactorial due to effects from both impaired kidney function and its causes and treatments for kidney disease impacting underlying vulnerability to severe respiratory disease and vaccine response [2]. These biological factors intersect with reduced ability to shield due to needing to attend hospital for specialist care, particularly those people treated with in-centre haemodialysis (IC-HD) [3]. While vaccination has greatly improved the relative risk of severe outcomes for many of the originally identified vulnerable groups, such as older individuals [4], it has offered modest gains for people receiving KRT [5].

For both transplant and dialysis populations, there is substantial evidence of attenuated responses to vaccinations against pre-pandemic pathogens [6, 7]. People receiving KRT were excluded from phase 3 severe acute respiratory syndrome coronavirus 2 (SARS-CoV-2) vaccine trials [8–10], but *in vitro* studies of immunogenicity of AZD1222 (Oxford–AstraZeneca) and BNT162b2 (Pfizer–BioNTech) showed reduced responses compared with people without kidney disease [11–14]. Therefore, despite vaccination, people receiving KRT remain at risk of severe illness from SARS-CoV-2 infection and are a population likely to have the most benefit from outpatient antiviral treatments. However, use of these medications in the KRT population is not straightforward. Paxlovid (nirmatrelvir/ritonavir), an oral antiviral, is contraindicated in the UK marketing authorisation for patients with severe kidney impairment or those receiving immunosuppressive drugs for kidney transplantation [15].

Randomised trials of sotrovimab, a neutralising monoclonal antibody (nMAb), had limited inclusion of patients receiving KRT [16]. There is also limited evidence for molnupiravir, an oral antiviral, among people receiving KRT [17, 18]. Nonetheless, in both England and Scotland, antiviral medications were pragmatically recommended for people receiving KRT. These were deployed via dedicated treatment centres [COVID Medicine Delivery Units (CMDUs)] in England and administered centrally via individual National Health Service (NHS) health boards in Scotland, both established in December 2021 to provide timely antiviral treatment of vulnerable patients in the community.

Due to the contraindications of Paxlovid, sotrovimab and molnupiravir are still the two commonly used treatment options for the KRT population in the UK. Therefore, it remains critical to understand the comparative effectiveness of sotrovimab and molnupiravir in preventing severe outcomes from COVID-19 in non-hospitalised patients receiving KRT. This is also especially relevant in the context of the ongoing global debate regarding the efficacy of sotrovimab. Although the World Health Organization and US Food and Drug Administration have recommended against the use of sotrovimab based on *in vitro* data [19, 20], the latest guidelines in the UK and several European countries still recommend its use [21].

In this study, we used two sources of high-quality routinely collected clinical data in England, the UK Renal Registry (UKRR) linked to the OpenSAFELY platform, to enable comprehensive clinical data and accurate identification of people receiving KRT to bridge this gap in knowledge for COVID-19 treatments during the Omicron era. In addition, to ensure generalisability of our results, we conducted a complementary analysis using data from the Scottish Renal Registry (SRR).

## MATERIALS AND METHODS

### Study population

#### OpenSAFELY-UKRR cohort

We included infected adults (≥18 years old) within the OpenSAFELY-TPP platform who were receiving KRT and had non-hospitalised treatment records for either sotrovimab or molnupiravir between 16 December 2021 and 1 August 2022, covering the Omicron waves where BA.1, BA.2 and BA.4/BA.5 were the predominant subvariants in England [22]. According to national guidance [15], these patients did not need hospitalisation for COVID-19 or new supplemental oxygen specifically for the management of COVID-19 symptoms when initiating the treatment. We focused on these two drugs because only a small number of infected patients on KRT were treated with Paxlovid [15], remdesivir or casirivimab/imdevimab. During the early part of the study (from 16 December 2021 to 10 February 2022) there was relative clinical equipoise between sotrovimab and molnupiravir, with either agent recommended for treatment of symptomatic high-risk patients in national guidance [23].

#### SRR cohort

All adults (≥18 years old) who were on KRT in Scotland who had a linked record for receiving either sotrovimab or molnupiravir between 21 December 2021 and 31 August 2022 were included.

### Data sources

#### OpenSAFELY-UKRR cohort

The dataset analysed within OpenSAFELY-TPP is based on 24 million people currently registered with general practitioner (GP) surgeries using TPP SystmOne software. All data were linked, stored and analysed securely within the OpenSAFELY platform (https://opensafely.org/). Data were pseudonymised and included coded diagnoses, medications and physiological parameters. No free-text data are included. All code is shared openly for review and reuse under an MIT open license (https://github.com/opensafely/sotrovimab-and-molnupiravir). Detailed pseudonymised patient data are potentially re-identifiable and therefore not shared. Primary care records are securely linked to the UKRR database, Office for National Statistics (ONS) mortality database, in-patient hospital records via the Secondary Uses Service (SUS), national coronavirus testing records via the Second Generation Surveillance System (SGSS) and the COVID-19 therapeutics dataset, derived from Blueteq software that CMDUs use to notify NHS England of COVID-19 treatments.

The UKRR database contains data from patients under secondary renal care. In this study we restricted our population to those in the UKRR 2021 prevalence cohort (i.e. patients alive and on KRT in December 2021).

#### SRR cohort

The SRR is a national registry of all patients receiving KRT in Scotland. It collates data from all nine adult renal units in Scotland and 28 satellite HD units serving a population of 5.4 million with 100% unit and patient coverage. Data on SARS-CoV-2 testing were obtained from the Electronic Communication of Surveillance in Scotland. Information on hospital admissions was obtained from the Scottish Morbidity Record and Rapid Preliminary Inpatient Data and data on deaths were obtained from the National Records of Scotland. Vaccination data were obtained from the Turas Vaccination Management Tool, which holds all vaccination records in Scotland. Data on treatment with sotrovimab or molnupiravir were obtained from information provided by the health boards to Public Health Scotland, in addition to data obtained via the Hospital Electronic Prescribing and Medicines Administration in the boards where available.

### Exposure

The exposure was treatment with sotrovimab or molnupiravir. In the OpenSAFELY-UKRR cohort, patients were excluded if they had treatment records of any other nMAbs or antivirals for COVID-19 before receiving sotrovimab or molnupiravir (*n* ≤ 5). Patients with treatment records of both sotrovimab and molnupiravir were censored at the start date of the second treatment (*n* = 8). In the SRR cohort, as the data linkage was only undertaken looking at sotrovimab or molnupiravir, we were unable to determine if any other antiviral treatments had been given prior to this.

### Outcomes

The primary outcome was COVID-19-related hospitalisation or COVID-19-related death within 28 days after treatment initiation. COVID-19-related hospitalisation was defined as hospital admission with COVID-19 as the primary diagnosis in the OpenSAFELY-UKRR cohort and defined as emergency hospital admission with COVID-19 as the main condition in the SRR cohort. COVID-19-related death was defined as COVID-19 being the underlying/contributing cause of death on death certificates in both cohorts.

Secondary outcomes were 28-day all-cause hospital admission or death and 60-day COVID-19-related hospitalisation/death. In the OpenSAFELY-UKRR cohort, to exclude events where patients were admitted in order to receive sotrovimab or other planned/regular treatment (e.g. dialysis), we did not count admissions coded as such or day cases detected by the same admission and discharge dates as hospitalisation events (Supplementary Table S1). Similarly, in the SRR cohort, only emergency hospital admissions with the length of hospital stay >0 were counted as outcome events.

### Statistical analyses

#### OpenSAFELY-UKRR cohort

Distributions of baseline characteristics were compared between the two treatment groups. Follow-up time of individual patients was calculated from the recorded treatment initiation date until the outcome event date, 28 days after treatment initiation, initiation of a second nMAb/antiviral treatment, death or patient deregistration date, whichever occurred first.

Risks of 28-day COVID-19-related hospitalisation/death were compared between the two groups using Cox proportional hazards models, with time since treatment as the time scale. The Cox models were stratified by NHS region to account for geographic heterogeneity in baseline hazards, with sequential adjustment for other baseline covariates. Model 1 was adjusted for age and sex; Model 2 additionally adjusted for high-risk cohort categories (solid cancer, haematological disease/stem cell transplant, immune-mediated inflammatory disorders or immunosuppression), KRT modality and years since KRT start date; Model 3 further adjusted for ethnicity, Index of Multiple Deprivation (IMD) quintiles, vaccination status and calendar date (with restricted cubic splines to account for non-linear effect); and Model 4 additionally adjusted for body mass index (BMI) category, diabetes, hypertension and chronic cardiac and respiratory diseases. Missing values of covariates were treated as separate categories. The proportional hazards assumption was tested based on the scaled Schoenfeld residuals.

As an alternative approach, we adopted the propensity score weighting (PSW) method to account for confounding bias. The covariates were balanced between the two drug groups through the average treatment effect (ATE) weighting scheme based on the estimated propensity scores. Balance check was conducted using standardised mean differences between groups (<0.10 as the indicator of well balanced). Robust variance estimators were used in the weighted Cox models.

Similar analytical procedures were used for secondary outcomes. In addition, we explored whether the following factors could modify the observed comparative effectiveness: KRT modality (dialysis or kidney transplantation), time period with different dominant variants (16 December 2021–15 February 2022 for BA.1, 16 February–31 May for BA.2 and 1 June–1 August for BA.4/BA.5) [22], BMI categories (≥30 versus <30 kg/m^2^), presence of diabetes, hypertension, chronic cardiac diseases or chronic respiratory diseases, days between testing positive and treatment initiation (<3 versus 3–5), age group (<60 versus ≥60 years), sex and ethnicity (White versus non-White).

Additional sensitivity analyses based on the stratified Cox models were conducted, including using complete case analysis or multiple imputation by chained equations to deal with missing values in covariates; using Cox models with calendar date as the underlying time scale to further account for temporal trends (and circulating variants); additionally adjusting for time between testing positive and treatment initiation, and time between last vaccination date and treatment initiation; additionally adjusting for rural–urban classification and other comorbidities and factors that might have influenced the clinician's choice of therapy through the patient's ability to travel to hospital for an infusion (learning disabilities, severe mental illness, care home residency or housebound status); using restricted cubic splines for age to further control for potential non-linear age effects; excluding patients with treatment records of both sotrovimab and molnupiravir or with treatment records of casirivimab/imdevimab, Paxlovid or remdesivir; excluding patients who did not have a positive SARS-CoV-2 test record before treatment or initiated treatment after 5 days since a positive SARS-CoV-2 test; creating a 1-day or 2-day lag in the follow-up start date to account for potential delays in drug administration; and conducting a cause-specific analysis for the 28-day COVID-19-related hospitalisation/death versus other hospitalisation/death.

#### SRR cohort

Similar statistical analyses were conducted in the SRR cohort, except where there was no relevant covariate information.

#### Exploratory analysis with untreated comparators in the OpenSAFELY-UKRR dataset

In addition to the comparative effectiveness analyses, following peer-review feedback we conducted an exploratory analysis to assess the effectiveness of sotrovimab and molnupiravir when compared with untreated COVID-19 patients on KRT. The comparator group was defined as patients in the UKRR 2021 prevalence cohort who had a COVID-19-positive test record between 16 December 2021 and 1 August 2022, not hospitalised on the date of a positive test and who did not receive any outpatient COVID-19 therapies in the following 28 days. In this analysis, the follow-up start date for both treated and untreated patients was the COVID-19-positive test date. Patients with a missing positive test date (or outside of the study period) were thus excluded from this exploratory analysis.

To account for immortal time bias (i.e. treated patients should not have outcome events between a positive test date and the treatment initiation date), we used a time-varying Cox model in which treated patients initially contributed person-time to the untreated group between positive test and treatment initiation and then contributed to the treated group after treatment initiation; untreated patients only contributed person-time to the untreated group after their positive test date. A robust variance estimator was used in this model. Similar covariate adjustment approaches were used as mentioned above.

## RESULTS

### OpenSAFELY-UKRR cohort

#### Patient characteristics

Between 16 December 2021 and 1 August 2022, a total of 2367 non-hospitalised COVID-19 patients on KRT were treated with sotrovimab (*n* = 1852) or molnupiravir (*n* = 515). The mean age of these patients was 55.9 years (SD 14.6), 43.5% were female, 85.4% being White and 92.6% having had three or more COVID-19 vaccinations. In the whole treated population, 69.6% were kidney transplant recipients and 30.4% were on dialysis. Among these, 81.8% of dialysis patients and 76.7% of transplant patients were treated with sotrovimab. Baseline characteristics were similar between the groups receiving different treatments (Table 1), but the sotrovimab group had a lower proportion of kidney transplant recipients and a higher proportion of patients with chronic cardiac disease. There were also some geographic variations in the prescription of these two drugs and greater use of molnupiravir earlier during the study period.

**Table 1:** Baseline characteristics of patients on KRT receiving molnupiravir or sotrovimab.

	OpenSAFELY-UKRR cohort			SRR cohort		
Characteristics	Molnupiravir group	Sotrovimab group	Total	Molnupiravir group	Sotrovimab group	Total
Patients, *n*	515	1852	2367	270	723	993
Age (years), mean (SD)	55.5 (14.6)	56.0 (14.6)	55.9 (14.6)	54.7 (12.7)	58.4 (14.2)	57.4 (13.9)
Female, *n* (%)	217 (42.1)	813 (43.9)	1030 (43.5)	113 (41.9)	310 (42.9)	423 (42.6)
White, *n* (%)	452 (87.9)	1567 (84.7)	2019 (85.4)			
Most deprived, *n* (%)	75 (14.9)	282 (15.7)	357 (15.6)	36 (13.3)	195 (27.0)	231 (23.3)
Region (NHS), *n* (%)						
East	144 (28.0)	489 (26.4)	633 (26.7)			
London	36 (7.0)	148 (8.0)	184 (7.8)			
East Midlands	35 (6.8)	357 (19.3)	392 (16.6)			
West Midlands	9 (1.8)	58 (3.1)	67 (2.8)			
North East	6 (1.2)	67 (3.6)	73 (3.1)			
North West	45 (8.7)	183 (9.9)	228 (9.6)			
South East	61 (11.8)	92 (5.0)	153 (6.5)			
South West	92 (17.9)	294 (15.9)	386 (16.3)			
Yorkshire	87 (16.9)	164 (8.9)	251 (10.6)			
KRT modality, *n* (%)						
Dialysis	131 (25.4)	588 (31.8)	719 (30.4)	21 (7.8)	324 (44.8)	345 (34.7)
Kidney transplant	384 (74.6)	1264 (68.3)	1648 (69.6)	249 (92.2)	399 (55.2)	648 (65.3)
Years since KRT start, median (IQR)	7 (4–13)	7 (4–12)	7 (4–13)	12 (4–18)	9 (2–14)	10 (3–15)
High-risk cohorts, *n* (%)						
Down syndrome	≤5	≤5				
Solid cancer	28 (5.4)	61 (3.3)	89 (3.8)			
Haematological disease	12 (2.3)	61 (3.3)	73 (3.1)			
Liver disease	≤5	29 (1.6)				
Immune-mediated inflammatory diseases	222 (43.1)	681 (36.8)	903 (38.2)			
Immunosuppression	17 (3.3)	57 (3.1)	74 (3.1)			
HIV/AIDS	≤5	6 (0.3)				
Rare neurological disease	≤5	6 (0.3)				
BMI (kg/m^2^), mean (SD)	28.4 (6.1)	28.3 (6.1)	28.3 (6.1)			
Comorbidities, *n* (%)						
Diabetes	189 (36.7)	710 (38.3)	899 (38.0)			
Chronic cardiac disease	111 (21.6)	506 (27.3)	617 (26.1)			
Hypertension	447 (86.8)	1585 (85.6)	2032 (85.9)			
Chronic respiratory disease	94 (18.3)	365 (19.7)	459 (19.4)			
Vaccination status, *n* (%)						
None	9 (1.8)	28 (1.5)	37 (1.6)	0 (0.0)	16 (2.2)	16 (1.6)
1–2	31 (6.0)	108 (5.8)	139 (5.9)	3 (1.1)	46 (6.4)	49 (4.9)
≥3	475 (92.2)	1716 (92.7)	2191 (92.6)	267 (98.9)	661 (91.4)	928 (93.5)
Days between test positive and treatment, median (IQR)	2 (1–3)	2 (1–3)	2 (1–3)	2 (1–2)	2(1–3)	2 (1–3)
Weeks between 16 December 2021 and treatment, median (IQR)	12 (4–17)	15 (8–22)	14 (7–21)	15 (8–26)	13 (9–23)	13 (9–24)
Primary renal diagnosis, *n* (%)						
Diabetes				34 (12.6)	129 (17.8)	163 (16.4)
Glomerulonephritis				71 (26.3)	164 (22.7)	235 (23.7)
Interstitial				113 (41.9)	219 (30.3)	332 (33.4)
Multisystem				32 (11.9)	115 (15.9)	147 (14.8)
Unknown (including missing)				20 (7.4)	96 (13.3)	116 (11.7)

In the OpenSAFELY-UKRR cohort, KRT start time, IMD, BMI, ethnicity and positive test date had 617, 73, 181, ≤5 and 199 missing values, respectively. In the SRR cohort, 17 postcodes did not match to an SIMD category and 12 primary renal diagnosis codes within the unknown group were missing.

#### Comparative effectiveness for the outcome events

Among the 2367 kidney patients treated with sotrovimab or molnupiravir, 38 cases (1.6%) of COVID-19-related hospitalisations/deaths were observed during the 28 days of follow-up after treatment initiation, with 21 (1.1%) in the sotrovimab group and 17 (3.3%) in the molnupiravir group; the number of COVID-19-related deaths was five or fewer in both groups.

**Figure 1:**
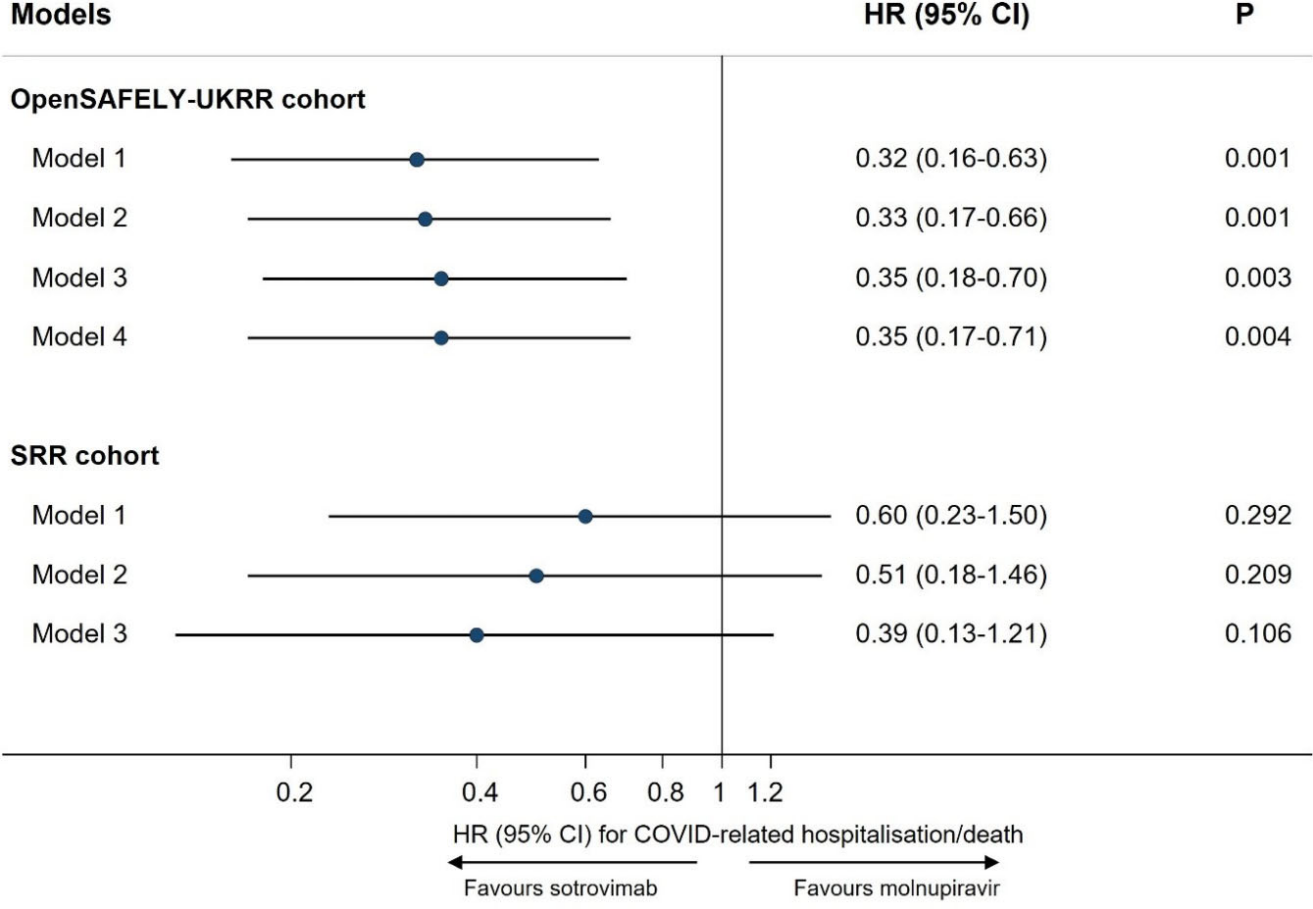
Comparing risk of 28-day COVID-19-related hospitalisation/death between sotrovimab versus molnupiravir in two cohorts. In the OpenSAFELY-UKRR cohort, Model 1 adjusted for age and sex; Model 2 additionally adjusted for high-risk cohort categories, KRT modality and duration; Model 3 additionally adjusted for ethnicity, IMD quintiles, vaccination status, calendar date; and Model 4 additionally adjusted for BMI category, diabetes, hypertension, chronic cardiac and respiratory diseases. In the SRR cohort, Model 1 adjusted for age and sex; Model 2 additionally adjusted for modality, PRD group and KRT duration; Model 3 additionally adjusted for SIMD, vaccination status and calendar time.

Results of stratified Cox regression showed that, after adjusting for multiple covariates, treatment with sotrovimab was associated with a substantially lower risk of 28-day COVID-19-related hospitalisation/death than treatment with molnupiravir [Model 4: HR 0.35 (95% CI 0.17–0.71); *P* = .004]. Consistent results favouring sotrovimab over molnupiravir were obtained from propensity score–weighted Cox models [Model 4: HR 0.39 (95% CI 0.19–0.80); *P* = .010], following confirmation of a successful balance of baseline covariates between groups in the weighted sample (Supplementary Table S2). The magnitude of HRs was stable during the sequential covariate adjustment process (ranging from 0.32 to 0.35 across different models; Fig. 1).

For the secondary outcomes, the analysis of 60-day COVID-19-related events revealed similar results in favour of sotrovimab (HRs ranging from 0.33 to 0.36; *P* < .05). For all-cause hospitalisations/deaths, 163 cases (6.9%) were observed during the 28 days of follow-up after treatment initiation [117 (6.4%) in the sotrovimab group and 46 (9.0%) in the molnupiravir group]. Results of stratified Cox regression showed a lower risk in the sotrovimab group than in the molnupiravir group (HRs ranging from 0.60 to 0.65 in Models 1–4; *P* < .05; Table 2).

**Table 2:** Comparing risks of non-COVID-specific outcomes between sotrovimab versus molnupiravir in two cohorts.

	OpenSAFELY-UKRR cohort			SRR cohort		
Outcomes	*N*/events	HR (95% CI) for sotrovimab (ref = molnupiravir)	*P*-value	*N*/events	HR (95% CI) for sotrovimab (ref = molnupiravir)	*P*-value
28-day all-cause hospitalisation/death	2350/163			993/75		
Model 1		0.65 (0.46–0.92)	.016		1.04 (0.61–1.76)	.879
Model 2		0.63 (0.44–0.89)	.010		0.80 (0.45–1.43)	.455
Model 3		0.62 (0.43–0.89)	.009		0.71 (0.39–1.29)	.273
Model 4		0.60 (0.41–0.85)	.004			
28-day other-cause hospitalisation/death	2350/130			993/56		
Model 1		0.85 (0.56–1.29)	.441		1.29 (0.68–2.40)	.426
Model 2		0.79 (0.52–1.21)	.276		0.97 (0.47–1.95)	.934
Model 3		0.76 (0.49–1.16)	.205		0.90 (0.44–1.84)	.776
Model 4		0.73 (0.48–1.12)	.151			

In the OpenSAFELY-UKRR cohort, Model 1 adjusted for age and sex; Model 2 additionally adjusted for high-risk cohort categories, KRT modality and duration; Model 3 additionally adjusted for ethnicity, IMD quintiles, vaccination status, calendar date; and Model 4 additionally adjusted for BMI category, diabetes, hypertension, chronic cardiac and respiratory diseases. In the SRR cohort, Model 1 adjusted for age and sex; Model 2 additionally adjusted for modality, PRD group and KRT duration; Model 3 additionally adjusted for SIMD, vaccination status and calendar time.

#### Sensitivity analyses and tests for effect modification

**Figure 2:**
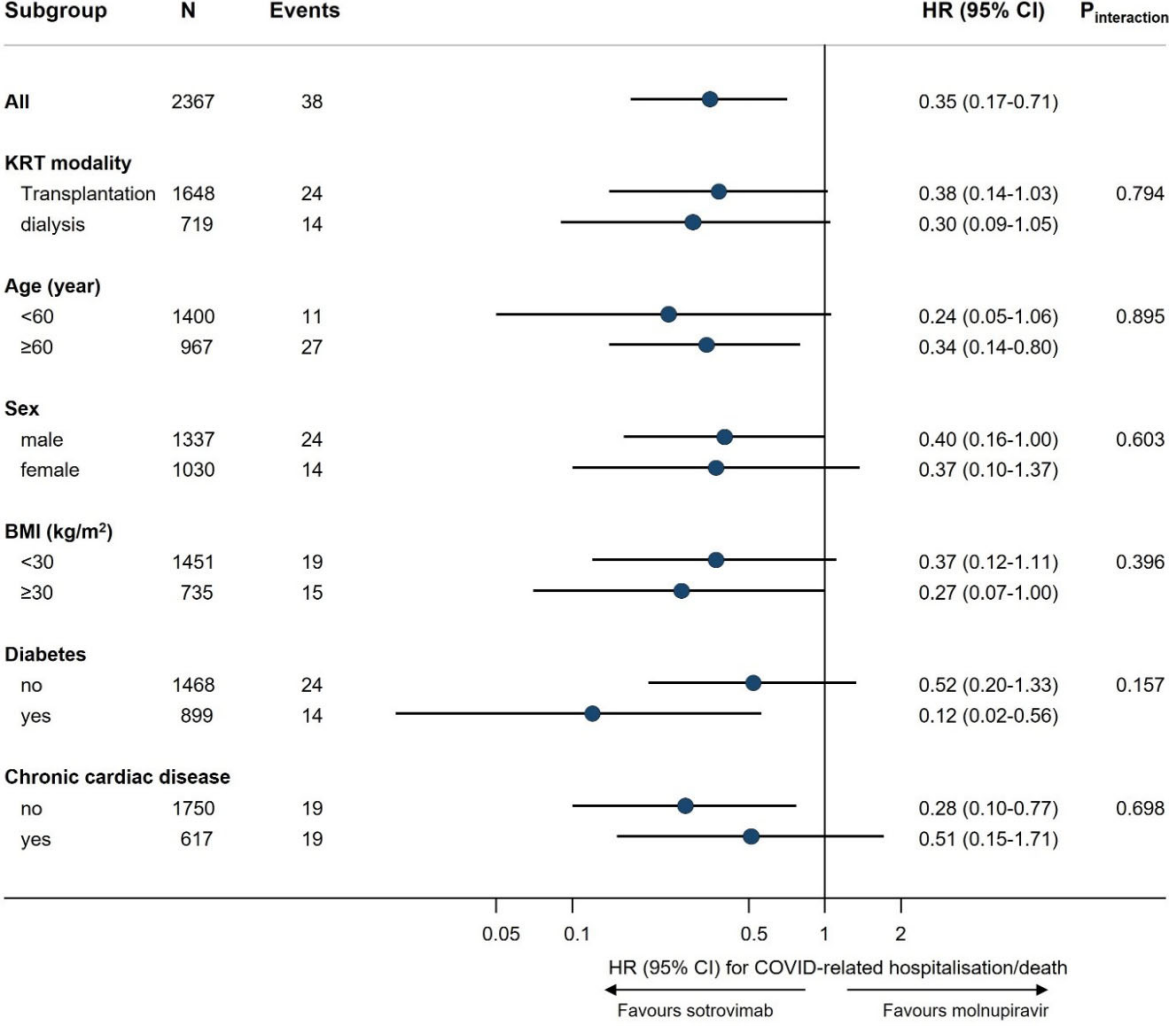
Subgroup analysis of sotrovimab versus molnupiravir in association with risk of 28-day COVID-19-related hospitalisation/death (OpenSAFELY-UKRR cohort). Subgroup analyses were based on the fully adjusted stratified Cox model (Model 4). *P*-value for interaction between drug group and each of the following variables was: time period 16 February 2022–31 May 2022 (0.577), time period 1 June 2022–1 August 2022 (0.640), hypertension (0.286), chronic respiratory diseases (0.449), days between test positive and treatment initiation (0.377) and White ethnicity (0.379), respectively; no analyses within each level of these variables were done because of a lack of sample size or outcome events within the subset of population.

Results of sensitivity analyses were consistent with the main findings (Supplementary Table S3). Among patients included in the cause-specific analysis (*n* = 2350), 33 had COVID-19-related hospitalisation/death and 130 had other hospitalisation/death events within 28 days after treatment initiation. The cause-specific Cox model showed that, unlike COVID-related outcomes, there was no evidence of an association of sotrovimab with other hospitalisation/death compared with molnupiravir (HRs ranging from 0.73 to 0.85 in Models 1–4; *P* > .05; Table 2) despite a greater number of events compared with the primary outcome. No substantial effect modification was observed in subgroup analyses (all *P*-values for interaction >0.10; Fig. 2).

#### Exploratory analysis with untreated patients as comparators

In the exploratory analysis on effectiveness, 4588 untreated COVID-19 patients on KRT were included as comparators to the two drug groups. Compared with sotrovimab and molnupiravir users, the untreated group was older [mean age 58.1 years (SD 15.9)], had a lower proportion of kidney transplant recipients (43.6%), being White (75.3%), having had three or more COVID-19 vaccinations (83.2%) and had a higher proportion of patients with chronic cardiac disease (33.8%; Supplementary Table S4).

Among the 4588 untreated patients, 224 cases (4.9%) of COVID-19-related hospitalisations/deaths were observed during the 28 days of follow-up after a positive test, among which there were 60 (1.3%) COVID-19-related deaths. Results of time-varying Cox regression showed that, after adjusting for multiple covariates, treatment with sotrovimab was associated with a substantially lower risk of 28-day COVID-19-related hospitalisation/death than no treatment [Model 4: HR 0.38 (95% CI 0.23–0.63); *P* < 0.001], but no significant association was observed for molnupiravir [Model 4: HR 0.81 (95% CI 0.44–1.49); *P* = .492; Table 3]. As for all-cause hospitalisations/deaths (520 cases in the untreated group), the sotrovimab group [Model 4: HR 0.78 (95% CI 0.62–0.98); *P* = .039] but not the molnupiravir group [Model 4: HR 1.07 (95% CI 0.75–1.54); *P* = .704] had a lower risk compared with the untreated group (Table 3).

**Table 3:** Comparing risks of outcome events within 28-days after a positive test between sotrovimab/molnupiravir versus untreated patients in the OpenSAFELY-UKRR dataset.

Outcomes	HR (95% CI) for sotrovimab (ref = untreated)	*P*-value	HR (95% CI) for molnupiravir (ref = untreated)	*P*-value
28-day COVID-19-related hospitalisation/death				
Model 1	0.38 (0.23–0.63)	<.001	0.94 (0.51–1.74)	.844
Model 2	0.33 (0.20–0.55)	<.001	0.79 (0.43–1.45)	.443
Model 3	0.38 (0.23–0.63)	<.001	0.79 (0.43–1.47)	.463
Model 4	0.38 (0.23–0.63)	<.001	0.81 (0.44–1.49)	.492
28-day all-cause hospitalisation/death				
Model 1	0.74 (0.59–0.92)	.008	1.01 (0.70–1.44)	.975
Model 2	0.74 (0.59–0.94)	.012	1.00 (0.70–1.44)	.995
Model 3	0.78 (0.61–0.98)	.037	1.05 (0.73–1.50)	.810
Model 4	0.78 (0.62–0.98)	.039	1.07 (0.75–1.54)	.704

In this analysis, 4588 untreated patients, 1624 sotrovimab users and 439 molnupiravir users were included (after excluding those whose positive test date was missing or outside of the study period). Model 1 adjusted for age and sex and stratified by region; Model 2 additionally adjusted for high-risk cohort categories, KRT modality and duration; Model 3 additionally adjusted for ethnicity, IMD quintiles, vaccination status, calendar date; and Model 4 additionally adjusted for BMI category, diabetes, hypertension, chronic cardiac and respiratory diseases.

### SRR cohort

Between 21 December 2021 and 31 August 2022, a total of 993 non-hospitalised COVID-19 patients on KRT were treated with sotrovimab (*n* = 723) or molnupiravir (*n* = 270). The mean age of these patients was 57.4 years (SD 13.9), 42.6% were female and 93.5% had three or more COVID-19 vaccinations; 65.3% were kidney transplant recipients and 34.7% were on dialysis. Compared with the molnupiravir group, the sotrovimab group had a lower proportion of kidney transplant recipients (55.2% versus 92.2%; Table 1).

During the 28 days of follow-up after treatment initiation, 19 cases (1.9%) of COVID-19-related hospitalisations/deaths were observed, with 12 (1.7%) in the sotrovimab group and 7 (2.6%) in the molnupiravir group. There were six COVID-19-related deaths in the sotrovimab group and five in the molnupiravir group.

Results of the Cox regression showed that after adjusting for age, sex, modality, primary renal diagnosis, Scottish IMD [24], vaccination status, KRT duration and calendar date, treatment with sotrovimab was consistent with a lower risk of 28-day COVID-19-related hospitalisation/death than treatment with molnupiravir, although CIs were broad and crossed the null [HR 0.39 (95% CI 0.13–1.21); *P* = .106; Fig. 1]. There was no substantial difference between sotrovimab and molnupiravir in the risk of all-cause hospitalisation/death (HRs ranging from 0.71 to 1.04 in Models 1–3; *P* > .05) or other hospitalisation/death (HRs ranging from 0.90 to 1.29 in Models 1–3; *P* > .05; Table 2).

## DISCUSSION

Our analysis shows that among people receiving KRT, treatment with sotrovimab is associated with a lower risk of severe outcomes from COVID-19 infection compared with molnupiravir during the Omicron wave in England in 2021–2022. We used a range of analytic methods to examine robustness of results and were able to carry out extensive adjustments for confounding given the availability of granular multisource real-world data. Analyses in an independent dataset from the SRR showed consistent effect estimates.

In addition, although it is likely that there are greater unmeasured differences in baseline health status and severity of COVID-19 between treated and untreated patients than between people treated with different medications (as reflected in Supplementary Table S4), results also showed supportive evidence for the effectiveness of sotrovimab but not molnupiravir when compared with no treatment in the infected KRT population.

This study used two validated KRT populations from 2021 reported by all kidney care centres in England and Scotland at the start of the Omicron outbreak in two independent analyses that gave broadly similar results. This is the first time analyses from the two independent renal registries, both recognised as high-quality and complete data sources, have been combined. The English data were combined with multisource data from the OpenSAFELY resource, which allowed extensive adjustment for confounding. The Scottish data had less statistical power and fewer granular variables for confounding adjustment and yielded more unstable point estimates across different statistical approaches (e.g. HR for 28-day COVID-19-related outcomes being 0.39 in the Cox regression and 0.78 in the propensity score analysis). Of note, in the English data, detailed adjustment for confounding did not materially change the results.

Several limitations of this study need to be considered. There are regional variations in terms of immune priming and survivorship bias in the KRT population because the pandemic has affected different parts of the country in different ways [25]. Similarly, there may be regional variations in how referral pathways operated for patients to receive antiviral treatment during the Omicron pandemic, which could underlie the marked regional variation of antiviral use in our data. To account for these differences, we stratified UKRR OpenSAFELY data analyses by English region and adjusted for region in propensity score analyses.

Despite the granular data on underlying health status, the possibility of residual confounding cannot be ruled out in this real-world observational study. In February 2022, prescribing guidelines changed and molnupiravir was deprioritised as a third-line treatment option [15], which reduces therapeutic equipoise and may make these two treatment groups less comparable. A pointer towards potential residual confounding may be the association between treatment and all-cause hospitalisation and death, which was not observed in the general population [26]. However, in this KRT population with high levels of comorbidity and frailty, it is possible that more effective treatment of COVID-19 reduced the incidence of other outcomes to an observable extent. Overall, given the size of the observed protective effect of sotrovimab and its robustness across multiple sensitivity analyses, residual confounding would have to be substantial to fully explain the findings. Consistent findings in independent validation in the SRR, where sources of bias and treatment pathways differed, adds further robustness to the analysis. In addition, a pragmatic trial for molnupiravir during the Omicron era, the UK PANORAMIC trial, showed that molnupiravir did not reduce the risk of hospitalisations/deaths among high-risk vaccinated adults with COVID-19 in the community [18].

Determining the current efficacy of treatments for COVID-19 is complicated due to changes in prevalence of circulating virus types, making gold-standard clinical trial data rapidly outdated. *In vitro* evidence is useful to understand activity of treatments against current viral subtypes but can be affected by the nature of assays and, in the case of sotrovimab, yield conflicting results [27–29]. Those data have led to changing and sometimes conflicting recommendations for prioritisation of COVID-19 treatments between countries and over time [19–21]. Well-conducted studies with routinely collected healthcare data can provide rapid analysis of drug effectiveness and safety and can be particularly valuable for populations underrepresented in clinical trials, such as KRT patients. In addition, analysis can be conducted within eras of different viral subtype dominance to update effectiveness estimates for new variants. Our data, alongside recent *in vitro* data, have helped to inform decision making about COVID-19 treatments, leading to a current recommendation for sotrovimab for people contraindicated for Paxlovid (including KRT patients) by the UK National Institute for Health and Care Excellence [21].

In summary, in routine care of non-hospitalised patients with COVID-19 on KRT, across periods of dominance of different subvariants of Omicron, sotrovimab was associated with a substantially lower risk of severe COVID-19 outcomes compared with molnupiravir.

## Supplementary Material

sfad184_Supplemental_File

## Data Availability

All data were linked, stored and analysed securely within the OpenSAFELY platform: https://opensafely.org/. Detailed pseudonymised patient data is potentially re-identifiable and therefore not shared.

## Notes

SRR: Formal ethical approval was waived according to Public Health Scotland Information Governance as analysis of routinely collected data. As the analysis used routinely collected and anonymized data, individual patient consent was not sought. Access and use of the data for the purpose of this work were approved following a Public Health Scotland Information Governance review of linking internal datasets. Only the Public Health Scotland analyst had access to the linked patient data, which could only be accessed via an NHS secure network.
