# Supplementary material for: Comparative effectiveness of sotrovimab and molnupiravir for preventing severe COVID-19 outcomes in patients on kidney replacement therapy: observational study using the OpenSAFELY-UKRR and SRR databases
Source: Clin Kidney J. 2023 Aug 29;16(11):2048–58. doi: 10.1093/ckj/sfad184 (PMC10616487; doi:10.1093/ckj/sfad184)
Supplement: sfad184_Supplemental_File [file sfad184_supplemental_file.docx]

**Supplementary material**

**Supplementary Table S1. Detected day case admissions that were not counted as hospitalisation events (OpenSAFELY-UKRR cohort).**

| **Admissions of which the admission and discharge dates were the same** | **Number of events** | |
| --- | --- | --- |
|  | **Molnupiravir** | **Sotrovimab** |
| Detected day cases on Day 0 (i.e., the treatment start date) | 23 | 105 |
| Those with MAbs procedure on Day 0 | 6 | 94 |
| Detected day cases on Day 1 | ≤5 | 11 |
| Those with MAbs procedure on Day 1 | ≤5 | 6 |
| Detected day cases on or after Day 2 | ≤5 | ≤5 |
| Those with MAbs procedure on or after Day 2 | ≤5 | ≤5 |

Note: The following admissions were not counted as hospitalisation events: (1) those recorded as “elective day case admission” or “regularadmission” in the patient classification field of SUS dataset, because such coding clearly indicates that the patient was admitted for a planned procedure or regular treatment (thus cannot represent severe COVID-19 outcomes); or (2) day cases that we additionally detected with the admission and discharge dates being the same. As shown in this table, most of those additionally detected day case admissions were for monoclonal antibody infusion procedure (i.e., admitted for receiving sotrovimab). Monoclonal antibody procedures were identified based on OPCS codes (X891, X892).

**Supplementary Table S2. Results of propensity score weighting analyses in both cohorts.**

| **Outcome** | **OpenSAFELY-UKRR cohort** | | | **SRR cohort** | | |
| --- | --- | --- | --- | --- | --- | --- |
|  | **N/Events** | **HR (95% CI) for sotrovimab (ref=molnupiravir)** | **P** | **N/Events** | **HR (95% CI) for sotrovimab (ref=molnupiravir)** | **P** |
| **28-day COVID-19 related hospitalisation/death** | 2367/38 |  |  | 993/19 |  |  |
| **Model 1** |  | 0.33 (0.16-0.64) | 0.001 |  | 0.61 (0.24-1.55) | 0.296 |
| **Model 2** |  | 0.35 (0.18-0.70) | 0.003 |  | 0.87 (0.33-2.20) | 0.784 |
| **Model 3** |  | 0.41 (0.20-0.83) | 0.013 |  | 0.78 (0.29-2.04) | 0.611 |
| **Model 4** |  | 0.39 (0.19-0.80) | 0.010 |  |  |  |

Note: In the balance check of both cohorts, standardised mean differences of all covariates between groups after weighting were <0.1.

**Supplementary Table S3. Sensitivity analysis of sotrovimab vs. molnupiravir in association with risk of 28-day COVID-19 related hospitalisation/death (OpenSAFELY-UKRR cohort).**

| **Sensitivity analyses** | **N** | **Events** | **HR (95% CI) for sotrovimab (ref=molnupiravir)** | **P** |
| --- | --- | --- | --- | --- |
| **Main analysis (for comparison purpose)** | 2367 | 38 | 0.35 (0.17-0.71) | 0.004 |
| **Additionally adjusting for days between test positive and treatment initiation, and months between last vaccination date and treatment initiation** | 2367 | 38 | 0.35 (0.17-0.72) | 0.004 |
| **Using Cox models with calendar date as the underlying time scale** | 2367 | 38 | 0.38 (0.17-0.82) | 0.015 |
| **Using restricted cubic splines for age** | 2367 | 38 | 0.35 (0.17-0.71) | 0.004 |
| **Additionally adjusting for rural-urban classification, comorbidities, care home and housebound status** | 2367 | 38 | 0.27 (0.13-0.56) | <0.001 |
| **Excluding patients with treatment records of both sotrovimab and molnupiravir, or any other therapies** | 2359 | redacted | 0.35 (0.17-0.71) | 0.004 |
| **Excluding patients without positive test record before treatment or initiated treatment after 5 days since positive test** | 2032 | 30 | 0.43 (0.19-0.96) | 0.040 |
| **Creating a 1-day lag in the follow-up start date** | 2356 | redacted | 0.29 (0.14-0.62) | 0.001 |
| **Creating a 2-day lag in the follow-up start date** | 2351 | 31 | 0.33 (0.15-0.73) | 0.006 |
| **Multiple imputation for covariates** | 2367 | 38 | 0.39 (0.19-0.79) | 0.009 |
| **Complete-case analysis** | 1580 | 24 | 0.38 (0.15-0.96) | 0.040 |

Note: HR=hazard ratio; CI=confidence interval. Sensitivity analyses were based on the fully-adjusted stratified Cox model (Model 4).

**Supplementary Table S4. Baseline characteristics of patients on KRT in the untreated group.**

| **Characteristics** | **Untreated group** | **Treated group (for comparison purpose)** |
| --- | --- | --- |
| **N** | 4588 | 2367 |
| **Age (year), mean (SD)** | 58.1 (15.9) | 55.9 (14.6) |
| **Female, n (%)** | 1717 (37.4) | 1030 (43.5) |
| **White, n (%)** | 3444 (75.3) | 2019 (85.4) |
| **Most deprived, n (%)** | 1212 (27.0) | 357 (15.6) |
| **Region (NHS), n (%)** |  |  |
| **East** | 999 (21.8) | 633 (26.7) |
| **London** | 348 (7.6) | 184 (7.8) |
| **East Midlands** | 774 (16.9) | 392 (16.6) |
| **West Midlands** | 308 (6.7) | 67 (2.8) |
| **North East** | 247 (5.4) | 73 (3.1) |
| **North West** | 370 (8.1) | 228 (9.6) |
| **South East** | 247 (5.4) | 153 (6.5) |
| **South West** | 560 (12.2) | 386 (16.3) |
| **Yorkshire** | 735 (16.0) | 251 (10.6) |
| **KRT modality, n (%)** |  |  |
| **Dialysis** | 2588 (56.4) | 719 (30.4) |
| **Kidney transplant** | 2000 (43.6) | 1648 (69.6) |
| **Years since KRT start, median (IQR)** | 6 (4-8) | 7 (4-13) |
| **High risk cohorts, n (%)** |  |  |
| **Solid cancer** | 91 (2.0) | 89 (3.8) |
| **Haematological disease** | 106 (2.3) | 73 (3.1) |
| **Immune-mediated inflammatory diseases** | 1299 (28.3) | 903 (38.2) |
| **Immunosuppression** | 93 (2.0) | 74 (3.1) |
| **BMI (kg/m^2^), mean (SD)** | 28.2 (6.3) | 28.3 (6.1) |
| **Comorbidities, n (%)** |  |  |
| **Diabetes** | 1951 (42.5) | 899 (38.0) |
| **Chronic cardiac disease** | 1551 (33.8) | 617 (26.1) |
| **Hypertension** | 3960 (86.3) | 2032 (85.9) |
| **Chronic respiratory disease** | 1035 (22.6) | 459 (19.4) |
| **Vaccination status, n (%)** |  |  |
| **None** | 244 (5.3) | 37 (1.6) |
| **One/two vaccinations** | 525 (11.4) | 139 (5.9) |
| **Three or more** | 3819 (83.2) | 2191 (92.6) |

Note: In the untreated group, KRT start time, IMD, BMI and ethnicity had 1334, 95, 359 and 13 missing values, respectively.
